# Early Insights on Mavacamten Usage in Canada: A Retrospective Cohort Study of the Mavacamten Patient Support Program

**DOI:** 10.1016/j.cjco.2026.02.012

**Published:** 2026-02-24

**Authors:** Kevin Ong, Patrick Garceau, Andrew M. Crean, Andrew D. Moeller, Lou Kolman, Stephanie Corriveau, Florence Brellier, Christina Wong, Michael R. Ward

**Affiliations:** a St-Paul’s Hospital, Vancouver, British Columbia, Canada; b Montreal Heart Institute, Montréal, Quebec, Canada; c University of Ottawa Heart Institute, Ottawa, Ontario, Canada; d Dalhousie University, Halifax, Nova Scotia, Canada; e Libin Cardiovascular Institute, Calgary, Alberta, Canada; f Bristol Myers Squibb, Montréal, Quebec, Canada; g Bristol Myers Squibb, Uxbridge, United Kingdom; h London Health Sciences Centre, London, Ontario, Canada

**Keywords:** hypertrophic cardiomyopathy, patient support program, mavacamten, real-world evidence, obstructive hypertrophic cardiomyopathy

## Abstract

**Background:**

Only a limited amount of real-world data are available on the demographic and clinical characteristics of adult patients with obstructive hypertrophic cardiomyopathy (oHCM) treated with mavacamten in Canada.

**Methods:**

This observational retrospective cohort study included adult patients with symptomatic oHCM enrolled in the mavacamten patient support program in Canada between January 4, 2023 and April 12, 2024. Baseline demographic information, clinical characteristics, medical history, and mavacamten treatment information were collected from the program database.

**Results:**

A total of 683 patients met the eligibility criteria for the study. The median age was 65.0 years (interquartile range: 57.0-73.0); 52.1% of the patients were male; and 67.1% were classified as being in New York Heart Association class II at baseline. Most patients were on beta-blocker monotherapy at baseline (63.7%) and the data cutoff point (76.3%). Almost all patients (99.4%) were initiated with 5 mg of mavacamten. The median follow-up time was 27.4 weeks (range: 1.6-70.1), and the median treatment duration was 24.6 weeks (range: 0.4-64.4). At the data cutoff point, 17.1% of patients were on 2.5 mg of mavacamten, 52.6% were on 5 mg, 20.2% were on 10 mg, and 2.2% were on 15 mg. Mavacamten was discontinued by 7.9% of patients, the main reasons being a side effect or adverse event (2.5%; 17 of 683), an undisclosed physician decision (1.8%; 12 of 683), or lack of efficacy (0.9%; 6 of 683).

**Conclusions:**

These early results provide the largest dataset on mavacamten utilization in the Canadian oHCM population. Baseline demographic and clinical characteristics and mavacamten dose distributions are comparable to those found in clinical trials and real-world studies.

**Clinical Trial Registration:**

NCT06549608.

## Central Illustration

**Figure:**
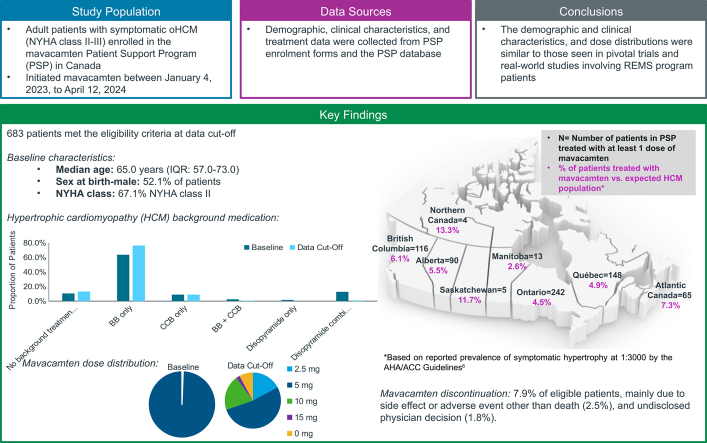

## Résumé

**Contexte:**

Il existe peu de données concrètes sur les caractéristiques démographiques et cliniques des patients adultes atteints de cardiomyopathie hypertrophique obstructive (CMHo) traités par mavacamten au Canada.

**Méthodologie:**

Il s'agit d'une étude de cohorte observationnelle rétrospective portant sur des patients adultes atteints de CMHo symptomatique inscrits au programme de soutien aux patients (PSP) avec prescription de mavacamten, au Canada, entre le 4 janvier 2023 et le 12 avril 2024. Les informations démographiques de base, les caractéristiques cliniques, les antécédents médicaux et les informations relatives au traitement par mavacamten ont été recueillies à partir de la base de données du PSP.

**Résultats:**

Au total, 683 patients répondaient aux critères d'éligibilité de l'étude. L'âge médian était de 65,0 ans (intervalle interquartile [IIQ] : 57,0-73,0), 52,1 % des patients étaient des hommes et 67,1 % étaient classés dans la classe fonctionnelle II de la NYHA au départ. La plupart des patients étaient sous monothérapie par Bêta-bloquant au départ (63,7 %) et à la fin de la collecte des données (76,3 %). Presque tous les patients (99,4 %) ont commencé le traitement avec 5 mg de mavacamten. La durée médiane du suivi était de 27,4 semaines (intervalle : 1,6-70,1) et la durée médiane du traitement était de 24,6 semaines (intervalle : 0,4-64,4). À la date limite de collecte des données, 17,1 % des patients recevaient 2,5 mg de mavacamten, 52,6 % recevaient 5 mg, 20,2 % recevaient 10 mg et 2,2 % recevaient 15 mg. Le mavacamten a été arrêté chez 7,9 % des patients, les principales raisons étant un effet secondaire ou un événement indésirable (2,5 %; 17/683), une décision médicale non précisée (1,8 %; 12/683) ou un manque d'efficacité (0,9 %; 6/683).

**Conclusions:**

Ces premiers résultats fournissent le plus grand jeu de données portant sur l'utilisation du mavacamten dans la population canadienne atteinte de CMHo. Les caractéristiques démographiques et cliniques de base et la distribution des doses de mavacamten sont comparables aux résultats des essais cliniques et des études en vie réelle.

Hypertrophic cardiomyopathy (HCM) is the most common genetic myocardial disease.^1^^,^^2^ Genetic testing in HCM identifies 2 distinct populations—those who are sarcomere mutation positive and those that are sarcomere mutation negative (causal gene mutation not identified and likely reflects polygenic disease)—with each population having inherent differences in phenotype and prognosis.^3^, ^4^, ^5^, ^6^ Mutations in the sarcomere protein alter myosin-actin cross-bridging, thereby triggering a sequence of molecular events that leads to a clinical phenotype that includes left ventricular hypertrophy, diastolic dysfunction, arrhythmia, and left ventricular outflow tract (LVOT) obstruction.^1^^,^^7^ Approximately 70% of patients with HCM have LVOT obstruction (peak gradient ≥ 30 mm Hg) at rest and/or after exercise, giving rise to exertional dyspnea, chest pain, fatigue, and presyncope/syncope.^8^ The prevalence of HCM and obstructive HCM (oHCM) in Canada has not been documented specifically.^9^ Prevalence estimates from other countries (US, United Kingdom, Germany, Japan) range from approximately 3.1-10 per 10,000 for HCM, and 1-5 per 10,000 for oHCM.^9^

Beta-blockers and non-dihydropyridine calcium-channel blockers are the first-line treatments for oHCM.^10^, ^11^, ^12^ Disopyramide, a potent negative inotrope, has been the second-line treatment for patients who do not respond to these first-line therapies, despite a paucity of data supporting its benefit.^12^, ^13^, ^14^ Disopyramide has a stronger ionotropic effect than beta-blockers and calcium-channel blockers and can be efficacious in a proportion of patients, but it does not directly target the molecular mechanisms underlying oHCM.^12^, ^13^, ^14^, ^15^ Invasive septal reduction therapy can be highly effective in reducing LVOT obstruction for patients who do not respond to pharmacologic therapy.^11^^,^^12^ However, invasive therapies may not be suitable for some patients (eg, older patients), are limited to availability at specialized centres with the necessary expertise, and carry a small risk of complications.^11^^,^^12^

Mavacamten is a first-in-class cardiac myosin inhibitor that was approved by the US Food and Drug Administration (FDA) in April 2022 for the treatment of adults with symptomatic oHCM (New York Heart Association [NYHA] class II-III).^16^^,^^17^ Unlike other pharmacologic treatments, mavacamten targets myosin-actin cross-bridging to reduce myocardial contractility and subsequently, LVOT obstruction.^11^^,^^16^ Mavacamten is safe to use in combination with beta-blockers or non-dihydropyridine calcium-channel blockers, but the safety profile of mavacamten in combination with disopyramide needs to be investigated further.^18^ Given that mavacamten can decrease the left ventricular ejection fraction (LVEF) and potentially induce heart failure, it is distributed to patients through the FDA-mandated risk evaluation mitigation strategy (REMS) program in the US.^16^ The REMS program mandates close monitoring of LVEF via echocardiograms, both prior to and during mavacamten treatment. Patients on mavacamten with an LVEF < 50% must temporarily discontinue treatment,^18^ possibly resuming it weeks later at a lower dose following LVEF recovery. Patients with an LVEF < 55% are advised against initiating mavacamten.^16^

Mavacamten was approved by Health Canada in November 2022 for the treatment of symptomatic oHCM (NYHA class II-III).^18^ This approval entailed fewer risk management requirements compared to the FDA-mandated REMS program. The requirement in Canada is for the distribution of a healthcare professional guide, patient guide, and patient card. Since January 4, 2023, mavacamten has been offered to patients in Canada through a patient support program (PSP). Patients enrolled in the PSP are assigned a nurse case manager who provides educational materials, coordinates prescriptions among prescribers and pharmacies, arranges echocardiogram assessments at external clinics, assists with reimbursement and financial support, and monitors treatment adherence.

Although the efficacy and safety of mavacamten has been demonstrated through several multicentre, randomized, placebo-controlled phase III clinical trials, including Mavacamten for Treatment of Symptomatic Obstructive Hypertrophic Cardiomyopathy (Clinical Study to Evaluate Mavacamten (MYK-461) in Adults With Symptomatic Obstructive Hypertrophic Cardiomyopathy [EXPLORER-HCM])^11^ and A Study to Evaluate Mavacamten in Adults with Symptomatic Obstructive HCM Who Are Eligible for Septal Reduction Therapy (VALOR-HCM),^19^ none of these large phase III trials were conducted in Canada. In addition, most real-world studies assessing the effectiveness and safety of mavacamten were conducted in the US and involved patients enrolled in the REMS program.^17^^,^^20^^,^^21^ As a result, a significant gap remains in the understanding of the real-world impact of mavacamten in Canada. This retrospective cohort study (NCT06549608) aims to bridge this gap by offering a comprehensive analysis of the demographic and clinical characteristics of patients initiating mavacamten in Canada. By elucidating dose regimens, titration patterns, and treatment durations, this study seeks to inform clinical practice. These findings will contribute to the growing body of evidence supporting the use of mavacamten for the treatment of symptomatic oHCM.

## Methods

### Study design and population

This was an observational, non-interventional, retrospective cohort study (NCT06549608) of adult patients with symptomatic oHCM who enrolled in the mavacamten PSP in Canada between January 4, 2023 and April 12, 2024. Patients were prescribed mavacamten by their treating physician from hospitals and institutions across Canada, and they were enrolled in the PSP (through completion of the PSP enrollment form by a healthcare professional). To be eligible for the study, patients were required to be ≥ 18 years of age, have initiated mavacamten as part of routine clinical care, and have consented to the use of their de-identified data generated from information collected through participation in the PSP. The study had no exclusion criteria. The index date was the date of PSP enrollment, and the date of mavacamten initiation was used for analyses related to treatment (eg, duration of mavacamten treatment). Patients received follow-up care until their discharge from the PSP or until the data cutoff (April 12, 2024), whichever was the earliest. The primary objective was to describe the demographic and clinical characteristics of the study population. The secondary objectives were to describe the dose regimen of mavacamten, its titration patterns, and the treatment duration.

### Ethics

The study was conducted in accordance with the International Society for Pharmacoepidemiology Guidelines for Good Pharmacoepidemiology Practices (GPP)^22^ and applicable regulatory requirements. The study protocol was approved by the Canadian SHIELD Ethics Review Board (observational study protocol CV027-1187). Only patients who consented to the use of their de-identified data were included in the study.

### Data sources and variables of interest

All baseline and follow-up data were collected from the PSP database (via Microsoft Customer Relationship Management [CRM]). Baseline data were collected prior to mavacamten initiation, and included demographic information, medical history, clinical characteristics, and mavacamten treatment information. Demographic data obtained included age, sex at birth, and geographic region. Medical history and clinical characteristics included oHCM treatment history at the time of mavacamten initiation, NYHA functional class, Valsalva-induced LVOT gradient (vLVOT), and LVEF. Continuous variables, such as age, vLVOT, and LVEF were collected as integers. The database required limited data cleaning, except for vLVOT and LVEF (for vLVOT: 6 observations with values ≥ 200 were considered implausible and replaced with missing; for LVEF: 11 entries were changed to missing—3 entries > 100% as they were considered implausible, and 8 entries with the text “ > 55%,” which could not be used for descriptive statistics).

The initiation date, dose, and dose regimen of mavacamten were collected at baseline and follow-up. The decision to titrate the dose was made by the physician, based on clinical need and their professional judgement, and in accordance with the Canadian product monograph. The dose regimen of mavacamten enabled the assessment of titration patterns and treatment duration. When applicable, the date of treatment discontinuation (last dose) was also captured. New patient information acquired through phone calls was recorded continuously in the PSP database throughout the patient’s enrollment. No data were collected after the discontinuation of mavacamten.

### Statistical analyses

The study did not test any formal hypotheses, and no formal sample size or statistical power calculations were performed. The sample size was based on the total number of patients enrolled in the mavacamten PSP in Canada who met study eligibility criteria at the data cutoff point.

Data analyses were conducted on all patients in the study population who received mavacamten treatment. All statistical analyses were descriptive. The median, range, and interquartile range (IQR) were examined for continuous variables, and counts and proportions were analyzed for categorical variables. Data analyses were performed, and the Sankey diagrams were created, using R software version 4.2.1 (R Foundation, Vienna, Austria).

Baseline demographic and clinical characteristics were summarized for all study patients. These characteristics also were summarized for patients on disopyramide at the time of mavacamten initiation, to determine how they differed from the rest of the PSP cohort. Additionally, baseline demographic and clinical characteristics were reported for patients with an order for 15 mg of mavacamten at any point during the study.

The follow-up duration was calculated from the time of PSP enrollment to the study data cutoff date for patients still enrolled in the PSP at this time, or to the date of last status for patients no longer in the PSP. The duration of mavacamten treatment was calculated from the date of mavacamten initiation to the date of discontinuation (last dose). Patients who had a record of mavacamten suspension lasting more than 1 month prior to the data cutoff were considered to have permanently discontinued mavacamten. If mavacamten was suspended because the patient was following the product monograph-recommended titration schedule, or due to delays in clinical management, the patient was not considered to have discontinued mavacamten permanently.

The number and proportion of patients on each dose regimen (2.5 mg, 5 mg, 10 mg, 15 mg) at mavacamten initiation and at the data cutoff point were reported. The proportion of patients on each dose regimen was calculated by dividing the number of patients on each dose by the total number of patients with available dosing information. The number and proportion of patients who experienced a dose up-titration (in mg: 2.5 to 5, 5 to 10, 10 to 15), down-titration (in mg: 15 to 10, 10 to 5, 5 to 2.5), or maintained their dose (ie, had the same dose as at the previous timepoint) following the initiation of mavacamten were reported. The proportions were calculated by dividing the number of patients with dose titration or maintenance by the total number of patients with available dose information. Mavacamten dose modifications were visualized using a Sankey diagram.

To align with phase III trials, such as EXPLORER-HCM, the dose distribution at 24 weeks was assessed for patients who remained on mavacamten for a minimum of 24 weeks (ie, the time from mavacamten initiation to the data cutoff point was at least 168 days). The dose distribution at 24 weeks was determined using the last dose ordered before the 24-week timepoint. The number of distinct doses ordered prior to the 24-week timepoint also was assessed.

The number of patients who reached a maintenance dose at least once was calculated for patients who had not discontinued mavacamten and had ≥ 4 months of follow-up care. A maintenance dose was defined as a dose that was maintained for ≥ 4 months.

Missing data were reported without imputation. The number of missing and nonmissing responses were reported for each variable of interest.

## Results

### Baseline demographics and clinical characteristics

**Figure 1:**
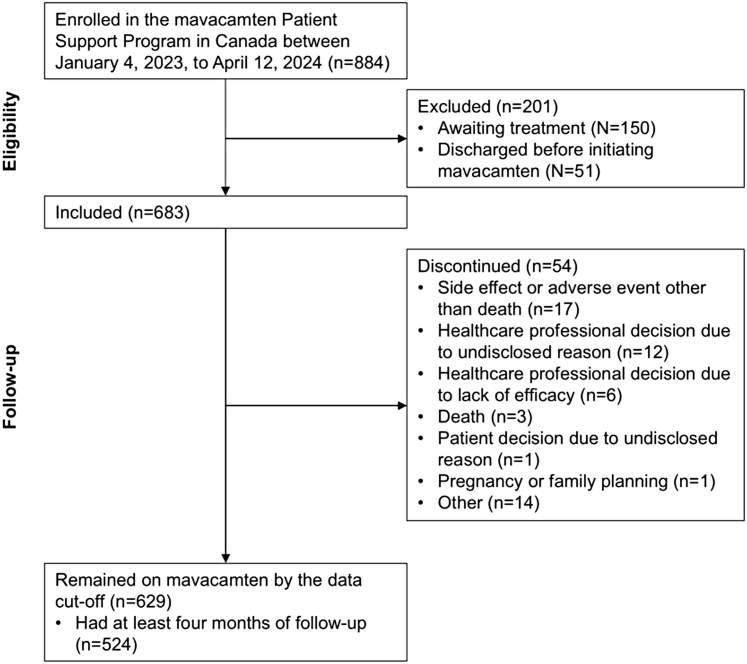
Consolidated standards of reporting trials (CONSORT) diagram showing flow of patients in this retrospective cohort study of the mavacamten patient support program in Canada.

A total of 884 patients were enrolled in the mavacamten PSP between January 4, 2023 and April 12, 2024 (Fig. 1), of which 683 (77.2%) met study criteria and were treated with at least one dose of mavacamten. The remaining 201 patients (22.7%) were excluded, as they had not initiated treatment by the data cutoff point; 51 were discharged before starting treatment, and 150 were still awaiting enrollment. The population “yet-to-be-treated” (N = 150) had characteristics similar to those of the study population, suggesting limited risk of selection bias (Supplemental Table S1).

Baseline patient demographics and clinical characteristics are summarized in Table 1. The median age of the patients was 65.0 years (IQR: 57.0-73.0), and 52.1% (356 of 683) were male. Most of the patients resided in Ontario (35.4%; 242 of 683), Quebec (21.7%; 148 of 683), British Columbia (17.0%; 116 of 683) and Alberta (13.2%; 90 of 683). Of the 683 patients included in this study, 458 (67.1%) were in NYHA class II. The median vLVOT gradient was 80.0 mm Hg (IQR: 62.0-102.0 mm Hg) and the median LVEF was 65.0% (IQR: 60.0%-70.0%).

**Table 1:** Baseline demographic and clinical characteristics at mavacamten initiation

Characteristics	Study population (N = 683)
**Age, Y**	65.0 (57.0–73.0)
**Male sex at birth**	356 (52.1)
**Canadian geographic region**	
Ontario	242 (35.4)
Quebec	148 (21.7)
British Columbia	116 (17.0)
Alberta	90 (13.2)
Manitoba	13 (1.9)
Saskatchewan	5 (0.7)
Atlantic Canada∗	65 (9.5)
Northern Canada†	4 (0.6)
**NYHA functional class**	
II	458 (67.1)
III	225 (32.9)
**Valsalva-induced LVOT gradient, mm Hg**	80.0 (62.0–102.0)
**LVEF (N = 672**‡**), %**	65.0 (60.0–70.0)

Values are n (%), or median (interquartile range).

LVEF, left ventricular ejection fraction; LVOT, left ventricular outflow tract; NYHA, New York Heart Association.

∗ Atlantic Canada included Nova Scotia, New Brunswick, Newfoundland and Labrador, and Prince Edward Island.

† Northern Canada included the Northwest Territories and Yukon.

‡ LVEF was considered missing for 11 patients. The LVEF for 8 patients was classified as undefined, and the LVEF for 3 patients had a value ≥ 100% (considered an entry error).

### Background HCM therapy at baseline and data cutoff point

**Figure 2:**
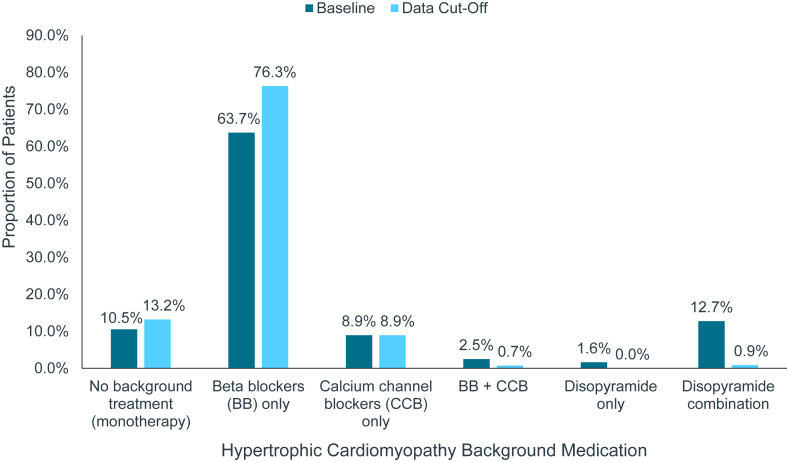
Hypertrophic cardiomyopathy background medication at baseline and at data cutoff point. (Baseline refers to medications taken prior to mavacamten initiation; data cutoff refers to the most recent data available for each patient prior to the end of the analysis period.) Proportion of patients on mavacamten monotherapy (no background medication), and those receiving mavacamten and beta-blockers (BBs) only, mavacamten and calcium-channel blockers (CCBs) only, mavacamten and dual combination of BBs + CCBs, mavacamten and disopyramide only, or mavacamten and disopyramide combination therapy (BBs and/or CCBs).

A mild increase occurred in the number of patients on mavacamten monotherapy, with 10.5% (72 of 683) receiving only mavacamten at the time of initiation and increasing to 13.2% (90 of 683) at the time of data cutoff (Fig. 2). Beta-blockers remained the most prevalent HCM therapy throughout the study, with 63.7% (435 of 683) receiving them at mavacamten initiation, with an increase to 76.3% (521 of 683) at the data cutoff point.

At baseline, 14.3% of patients (98 of 683) were treated with disopyramide, either alone or as adjunctive therapy (ie, in combination with beta-blockers and/or non-dihydropyridine calcium-channel blockers). The baseline demographic and clinical characteristics of these patients were assessed. The median age was 66.5 years (IQR: 56.5-74.0), and 56.1% of the patients (55 of 98) were male. Disopyramide utilization was highest in Ontario (43.9%; 43 of 98), British Columbia (32.7%; 32 of 98), and Quebec (12.2%; 12 of 98). Of the 98 patients on disopyramide at baseline, 65.3% (64of 98) were in NYHA class II, and 34.7% (34 of 98) were in NYHA class III. The median vLVOT gradient was 78.0 mm Hg (IQR: 66.3-100.0 mm Hg), and the median LVEF was 65.0% (IQR: 60.0%-69.3%). At the data cutoff point, only 0.9% of patients (6 of 683) remained on disopyramide combination therapy (ie, disopyramide in combination with mavacamten and beta-blockers and/or non-dihydropyridine calcium-channel blockers), with no patients on only disopyramide therapy.

### Mavacamten dose modification

Almost all patients included in the study (99.4%; 679 of 683) were initiated on 5 mg of mavacamten with a small fraction (0.6%; 4 of 683) started on 2.5 mg.

**Figure 3:**
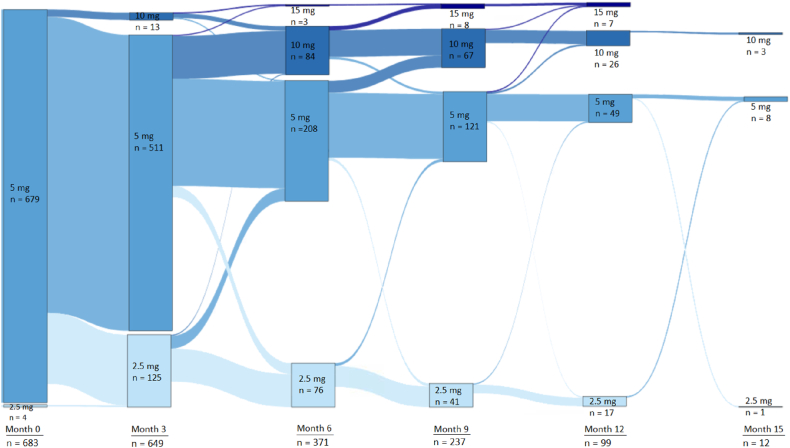
Mavacamten dose modification in Canadian patient support program (PSP) patients. Sankey diagram showing mavacamten dose status at different time points in Canadian PSP patients. A total of 679 patients were initiated at 5 mg of mavacamten per the Canadian product monograph. Treatment duration for each patient enrolled in the PSP varied depending on when they were included in this study prior to the date of data cutoff.

Mavacamten dose modifications among PSP patients in Canada are shown in Figure 3. The median follow-up time was 27.4 weeks (range: 1.6-70.1). Among the 683 patients treated with mavacamten, 175 (25.6%) required a dose reduction from 5 mg to 2.5 mg, with the median time to dose reduction being 10 weeks (IQR: 7.1-13.1). Interestingly, the time to dose change was longer for the 155 patients who switched from 5 mg to 10 mg, with a median of 17.7 weeks (IQR: 15.1-21.7). The median vLVOT gradient at baseline was slightly lower (although the IQR values have significant overlap) in patients whose treatment was down-titrated after the first dose: 75 mm Hg (IQR: 59.0-100) than in those whose treatment was up-titrated: 90.0 mm Hg (IQR: 72.0-108.0).

Of the patients who remained on mavacamten for a minimum of 24 weeks, 56.5% (226 of 400) were on 5 mg of mavacamten, 23.3% (93 of 400) were on 10 mg, and 20.3% (81 of 400) were on 2.5 mg at 24 weeks. No patient was on 15 mg of mavacamten at 24 weeks. At week 24, approximately half of the patients (50.3%; 201 of 400) remained on the same dose they were first prescribed; 41.8% (167 of 400) underwent one dose modification, 7.3% (29 of 400) had 2 dose modifications, and 0.8% (3 of 400) had 3 dose modifications.

At the data cutoff point, 52.6% of patients (359 of 683) were on 5 mg of mavacamten, 20.2% (138 of 683) were on 10 mg, 17.1% (117 of 683) were on 2.5 mg, and 2.2% (15 of 683) were on 15 mg. More than half of the patients (51.7%; 353 of 683) did not require a dose modification; 37.0% (253 of 683) had 1 dose modification, 9.5% (65 of 683) had 2 dose modifications, and 1.8% (12 of 683) had 3 dose modifications. Among the 629 patients who did not discontinue mavacamten, 49.6% (312 of 629) did not have a dose modification, 38.3% (241 of 629) had 1 dose modification, 10.3% (65 of 629) had 2 dose modifications, and 1.7% (11 of 629) had 3 dose modifications. The median number of distinct doses per patient was 1.0 (IQR: 1.0-2.0) for all patients, and 2.0 (IQR: 1.0-2.0) for patients who did not discontinue mavacamten.

A dose of 15 mg of mavacamten represents the maximum recommended daily dose.^18^ Baseline demographic and clinical characteristics were assessed for patients who remained on mavacamten for a minimum of 24 weeks and had an order for 15 mg of mavacamten at any point during the study (16 patients). At baseline, these patients had a median age of 58.0 years (IQR: 47.8-64.8); 50.0% were male; and 50.0% were in NYHA class II. The patients were mainly located in British Columbia (31.3%; 5 of 16), Alberta (25.0%; 4 of 16), Ontario (18.8%; 3 of 16), and Quebec (12.5%; 2 of 16). The median vLVOT gradient was 98.0 mm Hg (IQR: 74.3-111.3), and the median LVEF was 66.5% (IQR: 60.0%-70.0%) at baseline.

Of 524 patients who did not discontinue mavacamten and had at least 4 months of follow-up care, 314 (59.9%) reached a maintenance dose at least once. Among these 314 patients, 298 (94.9%) reached a maintenance dose only once, whereas 16 (5.1%) reached a maintenance dose more than once. A total of 196 patients (62.4%) reached a maintenance dose only once and had no record of another dose after achieving maintenance. For 148 of these 196 patients (75.5%), the maintenance dose was their initial dose. The remaining 48 patients (24.5%) had one or more other doses before reaching the maintenance dose, with the median time to the maintenance dose being 88 days (IQR: 63-109).

### Treatment duration and tolerability

Of the 683 patients treated with mavacamten, 54 (7.9%) discontinued mavacamten by the data cutoff point. The median treatment duration was 24.6 weeks (range: 0.4-64.4) for all patients included in the study, and 12.8 weeks (range: 0.4-48.7) for patients who discontinued mavacamten by the data cutoff point. The main reasons for discontinuation were a side effect or an adverse event other than death (2.5%; 17 of 683), undisclosed physician decision (1.8%; 12 of 683), and lack of efficacy (0.9%; 6 of 683). “Lack of efficacy” was determined and reported by the treating physician, based on their clinical judgment, and was not independently adjudicated by the study team. In patients who discontinued mavacamten because of an adverse event, the median treatment duration was 5.6 weeks (range: 0.4-21.1), showing that the adverse event causing the discontinuation occurred within 6 months for all of them. Unfortunately, details of these adverse events were not captured.

## Discussion

This retrospective cohort study presents the largest dataset on the demographic and clinical characteristics of patients initiating mavacamten for the treatment of symptomatic oHCM in Canada, as well as their mavacamten usage. The key findings of the study are summarized in the Central Illustration. At baseline, the study cohort was almost equally balanced by sex, had a median age of 65 years, and was well distributed in Canada, suggesting good adoption across the country. Because of the large number of patients included and the national coverage of the PSP, we believe that these study results can be generalized safely to Canadian patients receiving mavacamten. However, we cannot exclude the possibility that patients treated in the PSP might be more health-conscious, as early adopters of a new treatment. Following mavacamten initiation, most Canadian patients were maintained on their initial dose and required limited dose changes.

**Central Illustration:**
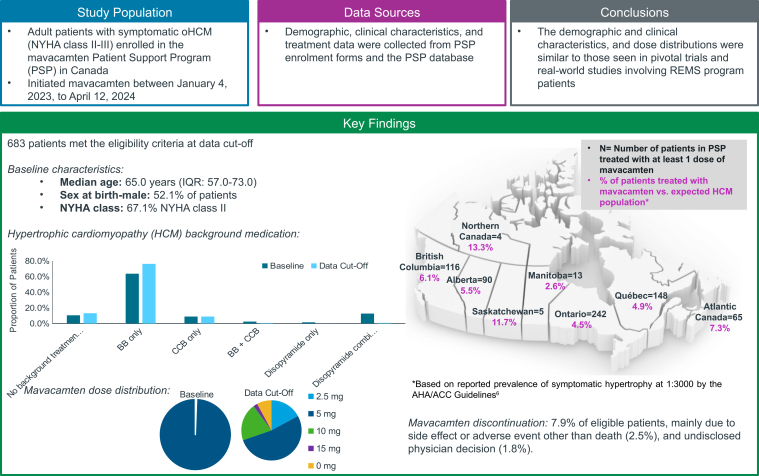
This retrospective cohort study presents the largest dataset on the demographic and clinical characteristics of patients initiating mavacamten for the treatment of symptomatic obstructive hypertrophic cardiomyopathy (oHCM) in Canada, as well as their mavacamten usage. ACC, American College of Cardiology; AHA, American Heart Association; BB, beta-blockers; CCB, calcium-channel blockers; HCM, hypertrophic cardiomyopathy; IQR, interquartile range; NYHA, New York Heart Association; oHCM, obstructive hypertrophic cardiomyopathy; PSP, patient support program; REMS, risk evaluation and mitigation strategy.

Baseline clinical characteristics observed in this study were comparable to those in other published data from clinical trials and real-world studies, with similar findings in echocardiographic parameters (vLVOT, LVEF) and background HCM medications (beta-blockers and calcium-channel blockers were the most commonly prescribed).^11^^,^^20^^,^^23^ However, key notable differences in age, sex, and NYHA classification were observed, highlighting the necessity for more Canadian real-world data.^11^^,^^17^^,^^20^^,^^23^ Specifically, patients enrolled in phase III clinical trials^11^^,^^23^ appeared to be younger than those in the current study cohort (around age 60 years vs age 65 years). In the US, the highest prevalence of oHCM is in patients aged 55-64 years.^24^^,^^25^ The slightly older age of the mavacamten PSP patients in Canada may suggest a delay in the recognition of symptomatic oHCM within routine clinical practice. Given Canada’s vast geography and dispersed population, geographic barriers likely contribute to challenges in accessing specialized care. Additionally, the limited number of HCM physicians and the relative scarcity of HCM specialists, especially compared to the US, may further contribute to delays in accessing clinical care for patients with oHCM. Although general practitioners and internists may identify oHCM appropriately, patients often face prolonged wait times before being seen at specialized oHCM centres.^26^ In addition to differences observed in age in this study and prior findings, we found that a majority of patients in phase III clinical trials were in NYHA class II (EXPLORER-HCM trial) or III (VALOR trial) at baseline, whereas most patients from the Canadian PSP included in this study were in NYHA class II at baseline (no patients were classified as being in NYHA class IV at baseline, per the mavacamten product label).^11^^,^^23^ Lastly, the majority of the patients in the REMS program were female (60.0%), whereas we found that close to half of PSP participants included in this study were male (52.1%).^17^^,^^20^ The female predominance is supported by prior data showing that, compared with men, women have a higher prevalence of the obstructive phenotype, worse diastolic function, and more severe heart failure symptoms at presentation.^27^ Overall, the differences between the US and Canadian healthcare systems may lead to different health-seeking behaviours and healthcare access. The results from this study underscore the need for more studies evaluating real-world mavacamten usage in Canada.

Despite the absence of RCT evidence of disopyramide’s efficacy in treating oHCM, current clinical guidelines continue to recommend it as a second-line treatment option for patients who do not respond to first-line therapies (eg, beta-blockers). The baseline utilization rate of disopyramide was higher in the Canadian PSP population (14.3%; 98 of 683) than in the US REMS population (5%; 3 of 66). The demographic and clinical characteristics of patients on disopyramide at baseline were no different than those of the overall patient population. The majority of patients receiving disopyramide were located in Ontario, British Columbia, and Quebec, which may be attributable to the availability of larger HCM programs and the wider availability of the drug in these regions. The proportion of patients receiving mavacamten and disopyramide in this study was negligible by the time of data cutoff. This trend aligns with the Canadian product monograph for mavacamten, which advises against concomitant use of mavacamten with disopyramide due to additive negative inotropic effects.^18^ Patients enrolled in the EXPLORER-HCM clinical trial were not permitted to continue taking disopyramide with mavacamten treatment but could continue other background HCM therapies.^11^ Similarly, patients in the REMS program are required to discontinue disopyramide prior to starting mavacamten,^21^ or to gradually transition off disopyramide while initiating mavacamten.^28^ In contrast, patients in the VALOR-HCM trial were permitted to continue disopyramide while initiating mavacamten treatment.^23^ The inclusion of patients on both disopyramide and mavacamten in VALOR-HCM suggests that not all physicians considered it necessary to immediately discontinue disopyramide upon mavacamten initiation.^23^ In contrast to disopyramide use while receiving mavacamten treatment, the proportion of patients receiving mavacamten while also taking beta-blockers in this study was found to be higher at the data cutoff point, compared to baseline. This increased use of beta-blockers in combination with mavacamten treatment may be attributed to the use of beta-blockers as a replacement rate-control strategy after discontinuing disopyramide, as recommended by the product monograph. Previous findings have shown that beta-blocker use in combination with mavacamten may blunt the full therapeutic effect of mavacamten, particularly with respect to exercise capacity.^29^

The dosing patterns observed in this study were comparable to findings from other real-world studies.^17^^,^^20^ Almost all patients were started on 5 mg of mavacamten, and a majority (52.6%) remained on the same dose at the data cutoff point. Similar proportions of patients were down-titrated to 2.5 mg or up-titrated to 10 mg of mavacamten throughout the study, with few patients requiring the 15-mg dose. As most patients were not followed in this study for > 24 weeks (the median treatment duration was 24.6 weeks), only a small proportion of patients likely were observed to be treated with 15 mg of mavacamten, as a majority of patients were not followed for a long enough period in this study to determine if they required up-titration to 15 mg. Following the stepwise dosage adjustment recommendation outlined in the product monograph, a period of > 24 weeks is required to be eligible for the 15-mg dose.^18^ Hence, full efficacy likely cannot be expected in this cohort. Similar to these results, Desai et al. followed 6299 patients enrolled in the first 22 months of the REMS program, and found that almost all patients (97.2%; 6176 of 6354) were started on 5 mg of mavacamten.^17^ Among the 3228 patients who completed 6 months of follow-up, most (74.1%) were on 5 mg or 10 mg of mavacamten; 20.4% were on 2.5 mg, and 5.6% were on 15 mg.^17^ Likewise, Kim et al. followed 50 patients for an average of 36 weeks following mavacamten initiation, and they reported that 66.7% of patients were on 5 or 10 mg, whereas 20.0% were on 2.5 mg, and 13.3% were on 15 mg at their most recent follow-up.^20^ Conflicting results have been reported regarding mavacamten dose adjustments in real-world studies, which may be attributed to differences in the clinical characteristics observed at baseline.^30^^,^^31^ In our study, less than half of the patients required at least one dose modification. Abood et al. reported that no patients required a dose adjustment during the 12-week treatment period.^31^ Meanwhile, Scholtz et al. reported that more than 50% of symptomatic oHCM patients required a dose adjustment after 12 weeks of treatment, with a higher mean provoked LVOT gradient observed at baseline than what was observed by Abood et al.^30^^,^^31^ Collectively, findings from our study and other real-world studies based on REMS program data demonstrate that a majority of patients were able to achieve symptom improvement at dosages of 5 mg or 10 mg of mavacamten, and that most patients did not require multiple dosage modifications to attain clinical benefit. Given that echocardiograms are essential for monitoring and guiding mavacamten treatment, the limited number of dose modifications observed in this study could mean reduced healthcare resource utilization costs associated with mavacamten treatment. Although this possibility was not assessed directly in the current study, it represents an important consideration for future research.

In our study, the median vLVOT gradient of patients receiving 15 mg of mavacamten at any point during the observation period was found to be higher than that of the overall patient population. These results are expected, as a higher vLVOT gradient (≥ 30 mm Hg) and symptom persistence may necessitate titration to a higher dose of mavacamten.^30^ The recommendation per the product monograph is to up-titrate in a stepwise fashion (ie, from 5 mg to 10 mg, then 10 mg to 15 mg) if LVEF ≥ 55% and vLVOT ≥ 30 mm Hg after 12 weeks of mavacamten treatment.^18^

The median treatment duration observed in this study (24.6 weeks; 5.7 months) was shorter than that previously reported.^21^ In their study, Ramonfaur and colleagues reported a median treatment duration of 9 months for their prospective observational cohort study of oHCM patients prescribed mavacamten during an 18-month study period (July 7, 2022 to January 6, 2024).^21^ The treatment duration observed in the current study was limited by both the eligibility period (15 months; January 4, 2023 to April 12, 2024) and the speed at which patients were able to initiate treatment. Delays in navigating insurance coverage and scheduling frequent echocardiograms in a resource-constrained healthcare system can contribute to delayed treatment initiation in Canada.

A higher discontinuation rate (7.9%) was found in this study, compared to that observed in other real-world studies and the EXPLORER-LTE trial. Desai et al. reported that no patients required permanent discontinuation of mavacamten over the 22-month study period.^17^ Kim et al. found that only 3 patients (6.0%) permanently discontinued mavacamten (due to fatigue and/or malaise or loss of insurance coverage).^20^ These studies were conducted in the US, and the close monitoring of patients required as part of the REMS program may have allowed for early detection and management of adverse events or side effects without requiring permanent discontinuation. Similarly, a discontinuation rate of 5.6% was reported in the EXPLORER-LTE trial due to treatment-emergent adverse events. Conversely, routine clinical care in Canada requires less patient monitoring. Mavacamten was approved in Canada with a less-stringent risk management plan involving only the distribution of educational materials, including a healthcare professional guide, a patient guide, and a patient card. Cytochrome P (CYP) testing was not implemented routinely in Canada. The relatively higher rate of discontinuation observed in our study indicates a preference for Canadian physicians to discontinue mavacamten when patients experience side effects or adverse events, or for other undisclosed reasons. Future research should focus on collecting more information regarding the safety of mavacamten (eg, number of adverse events and relatedness to treatment) within the Canadian clinical practice setting.

The limitations of the study include those that are inherent to retrospective data collection, such as nonstandardized follow-up intervals. The data source (PSP database) may have missing, incomplete, or inaccurate data, and variability in the availability of data by region may be present. Of note, the race and/or ethnicity of the patients was not collected in this study. This is a limitation, given that mavacamten is metabolized by the CYP2C19 enzyme, and racial differences in CYP2C19 genotype status are known to exist.^18^ Another limitation of the study is that the dosing data were obtained from order information documented through the PSP, and this was not confirmed by the patients. Lastly, the study captured mavacamten treatment information, but this was not correlated with clinical outcomes (eg, changes in LVOT, LVEF, or NYHA class).

## Conclusion

The baseline demographic and clinical characteristics, background HCM therapy, and dose regimen of patients receiving mavacamten treatment through enrollment in the Canadian PSP program are comparable to data reported from clinical trials and real-world studies conducted outside of Canada. The majority of patients in this study were initiated on 5 mg of mavacamten and required limited dose changes. This study presents the first real-world Canadian dataset on oHCM patients treated with mavacamten and highlights the need for additional data evaluating clinical outcomes of symptomatic oHCM patients treated with mavacamten within the Canadian clinical practice setting.
