# Supplementary material for: Early Insights on Mavacamten Usage in Canada: A Retrospective Cohort Study of the Mavacamten Patient Support Program
Source: CJC Open. 2026 Feb 24;8(6):627–36. doi: 10.1016/j.cjco.2026.02.012 (PMC13282525; doi:10.1016/j.cjco.2026.02.012)
Supplement: Supplementary Material [file mmc1.docx]

**Supplementary material**

**Supplemental Appendix S1. STROBE Statement—Checklist of items that should be included in reports of cohort studies and adherence check for each recommendation (last column)**

|  | Item No | Recommendation |  |
| --- | --- | --- | --- |
| **Title and abstract** | 1 | (*a*) Indicate the study’s design with a commonly used term in the title or the abstract | **x** |
|  |  | (*b*) Provide in the abstract an informative and balanced summary of what was done and what was found | **x** |
| Introduction | | |  |
| Background/rationale | 2 | Explain the scientific background and rationale for the investigation being reported | **x** |
| Objectives | 3 | State specific objectives, including any prespecified hypotheses | **x** |
| Methods | | |  |
| Study design | 4 | Present key elements of study design early in the paper | **x** |
| Setting | 5 | Describe the setting, locations, and relevant dates, including periods of recruitment, exposure, follow-up, and data collection | **x** |
| Participants | 6 | (*a*) Give the eligibility criteria, and the sources and methods of selection of participants. Describe methods of follow-up | **x** |
|  |  | (*b*) For matched studies, give matching criteria and number of exposed and unexposed | **N.A.** |
| Variables | 7 | Clearly define all outcomes, exposures, predictors, potential confounders, and effect modifiers. Give diagnostic criteria, if applicable | **x** |
| Data sources/ measurement | 8* | For each variable of interest, give sources of data and details of methods of assessment (measurement).  Describe comparability of assessment methods if there is more than one group | **x**  **N.A.** |
| Bias | 9 | Describe any efforts to address potential sources of bias | **x** |
| Study size | 10 | Explain how the study size was arrived at | **x** |
| Quantitative variables | 11 | Explain how quantitative variables were handled in the analyses. If applicable, describe which groupings were chosen and why | **x** |
| Statistical methods | 12 | (*a*) Describe all statistical methods, including those used to control for confounding | **x** |
|  |  | (*b*) Describe any methods used to examine subgroups and interactions | **N.A.** |
|  |  | (*c*) Explain how missing data were addressed | **x** |
|  |  | (*d*) If applicable, explain how loss to follow-up was addressed | x |
|  |  | (*e*) Describe any sensitivity analyses | **N.A.** |
| Results | | |  |
| Participants | 13* | (a) Report numbers of individuals at each stage of study—eg numbers potentially eligible, examined for eligibility, confirmed eligible, included in the study, completing follow-up, and analysed | **x** |
|  |  | (b) Give reasons for non-participation at each stage | **x** |
|  |  | (c) Consider use of a flow diagram | **x** |
| Descriptive data | 14* | (a) Give characteristics of study participants (eg demographic, clinical, social) and information on exposures and potential confounders | **x** |
|  |  | (b) Indicate number of participants with missing data for each variable of interest | **x** |
|  |  | (c) Summarise follow-up time (eg, average and total amount) | **x** |
| Outcome data | 15* | Report numbers of outcome events or summary measures over time | **x** |
| Main results | 16 | (*a*) Give unadjusted estimates and, if applicable, confounder-adjusted estimates and their precision (eg, 95% confidence interval).  Make clear which confounders were adjusted for and why they were included | **x**  **N.A.** |
|  |  | (*b*) Report category boundaries when continuous variables were categorized | **N.A.** |
|  |  | (*c*) If relevant, consider translating estimates of relative risk into absolute risk for a meaningful time period | **N.A.** |
| Other analyses | 17 | Report other analyses done—eg analyses of subgroups and interactions, and sensitivity analyses | **x** |
| Discussion | | |  |
| Key results | 18 | Summarise key results with reference to study objectives | **x** |
| Limitations | 19 | Discuss limitations of the study, taking into account sources of potential bias or imprecision. Discuss both direction and magnitude of any potential bias | **x** |
| Interpretation | 20 | Give a cautious overall interpretation of results considering objectives, limitations, multiplicity of analyses, results from similar studies, and other relevant evidence | **x** |
| Generalisability | 21 | Discuss the generalisability (external validity) of the study results | **x** |
| Other information | | |  |
| Funding | 22 | Give the source of funding and the role of the funders for the present study and, if applicable, for the original study on which the present article is based | **x** |

N.A. Not Applicable

*Give information separately for exposed and unexposed groups.

**Supplemental Table S1. Baseline Characteristics of study population and PSP enrollees excluded because pending treatment**

| **Characteristics** | **Study Population (N=683)** | **PSP enrollees excluded because pending treatment (N=150)** |
| --- | --- | --- |
| **Age (years), median (IQR)** | 65.0 (57.0-73.0) | Unknown (age to be captured at treatment start) |
| **Male sex at birth, n (%)** | 356 (52.1) | 81 (54.0) |
| **Canadian geographic region, n (%)** |  |  |
| Ontario | 242 (35.4) | 34 (22.7) |
| Quebec | 148 (21.7) | 29 (19.3) |
| British Columbia | 116 (17.0) | 52 (34.7) |
| Alberta | 90 (13.2) | 24 (16.0) |
| Manitoba | 13 (1.9) | 6 (4.0) |
| Saskatchewan | 5 (0.7) | 0 (0) |
| Atlantic Canada^a^ | 65 (9.5) | 5 (3.3) |
| Northern Canada | 4 (0.6) | 0 (0) |
| **NYHA functional class*, n (%)** |  |  |
| NYHA class II | 458 (67.1) | 60 (60.6) |
| NYHA class III | 225 (32.9) | 39 (39.4) |
| **Valsalva-induced LVOT gradient* (mmHg), median (IQR)** | 80.0 (62.0-102.0) | 84.0 (60.0-109.0) |
| **LVEF* (%), median (IQR)** | 65.0 (60.0-70.0) | 65.0 (60.0-70.0) |
| ^c^LVEF was considered missing for 11 patients in total (including 3 for the down-titration group and 1 for the up-titration group). | | |
| * in untreated patients, 51 missing values for NYHA class, 49 for LVOT and 51 for LVEF | | |
